# Prevalence of electrolyte disturbances in perinatal asphyxia: a prospective study

**DOI:** 10.1186/s13052-018-0496-7

**Published:** 2018-05-21

**Authors:** Jitendra Thakur, Nisha Keshary Bhatta, Rupa Rajbhandari Singh, Prakash Poudel, Madhab Lamsal, Anjum Shakya

**Affiliations:** 1 Department of Pediatrics and Adolescent Medicine, BPKIHS, Dharan, Nepal; 2 Division of Neonatology, Department of Pediatrics and Adolescent Medicine, BPKIHS, Dharan, Nepal; 3 Department of Biochemistry, BPKIHS, Dharan, Nepal; 4 Department of Pediatrics and Adolescent Medicine, Patan Academy of Health Sciences, Kathmandu, Nepal

**Keywords:** Asphyxia, Hyponatremia, Hyperkalemia, Hypocalcaemia

## Abstract

**Background:**

Birth asphyxia is defined as the presence of hypoxia, hypercapnia, and acidosis leading the newborn to systemic disturbances probably electrolyte disturbance also. Knowledge of these electrolyte disturbances is very valuable as it can be an important parameter affecting perinatal morbidity, mortality and ongoing management.

**Methods:**

Serum sodium, potassium and ionized calcium of asphyxiated term newborn were sent within one hour of birth as per the inclusion criteria. Statistical comparison of mean values of different electrolytes between different groups of perinatal asphyxia was performed by ANOVA test for parametric data and significant data were further analyzed using post hoc test. Bivariate analysis was done to determine the correlations between Apgar score at 5 min and serum electrolytes. Pearson test was used to calculate the correlation coefficient. Box plot was used to show the median and quartile between serum electrolytes and Apgar score at 5 min.

**Result:**

The mean values of sodium for mild, moderate and severe asphyxia were 135.52, 130.7 and 127.15 meq/l respectively. The values of potassium for mild, moderate and severe asphyxia were 4.96, 5.93 and 6.78 meq/l respectively. Similarly, the mean values of ionized calcium for mild, moderate and severe asphyxia were 1.07, 1.12 and 0.99 mmol/l respectively. The values of sodium and potassium among different severity of asphyxia were significantly different (*p*-value< 0.001). Significant positive correlation was found between serum sodium and Apgar score at 5 min. Significant negative correlation was present between serum potassium and Apgar score at 5 min.

**Conclusion:**

The degree of hyponatremia and hyperkalemia was directly proportional to the severity of birth asphyxia. So these electrolyte disturbances should always be kept in mind while managing cases of perinatal asphyxia and should be managed accordingly.

## Background

In basic term birth asphyxia is delay in establishing spontaneous respiration upon delivery of a newborn [1]. More precisely, birth asphyxia is defined as the presence of hypoxia, hypercapnia, and acidosis leading to systemic disturbances in the newborn [2]. As per AAP (American Academy of Pediatrics) and ACOG (American College of Obstetrics and Gynecology), all the following must be present for designation of asphyxia such as, profound metabolic or mixed acidemia (pH < 7) in cord, Persistence of APGAR scores 0–3 for longer than 5 min, neonatal neurological sequel (eg: seizures, coma, hypotonia) and multiple organ involvement (kidney, lungs, liver, heart, intestine) [3]. It is a common problem with the incidence varying from 0.5–2% of live births [4]. Some report the incidence from 1 to 8 per 1000 live births [5].

Normally hypernatremia is expected in the early neonatal period as there is contraction of extracellular fluid due to excretion of water through kidney and high insensible water loss whereas in neonates with perinatal asphyxia there might be hyponatremia as there is increased secretion of anti-diuretic hormone (ADH) in neonates with HIE which leads to increased water retention and hence dilutional hyponatremia [6]. The other reason for hyponatremia is that the capacity of sodium reabsorption is limited and if the load of sodium reaching the Collecting Tubules (CT) increases significantly, reabsorption does not occur proportionately and the sodium load is excreted in the urine [7]. Other contributing factors to hyponatermia is partial resistance to aldosterone [8].

In newborns there is hyperkalemia in early neonatal period due to shift of potassium from the intracellular to extracellular space. The magnitude of this shift roughly correlates with the degree of immaturity i.e. the more premature the baby the more chance of hyperkalemia [9]. Serum potassium subsequently falls as this internal potassium “load” is excreted by the kidneys [10]. Whereas the rise in level of serum potassium can be explained from the fact that birth asphyxia is associated with acidosis, and in metabolic acidosis, more than one-half of the excess hydrogen ions are buffered in the cells. In this setting, electro neutrality is maintained in part by the movement of intracellular potassium into the extracellular fluid. It can also be due to acute renal failure secondary to birth asphyxia which leads to decreased excretion of potassium and hence hyperkalemia.

In normal newborn total calcium concentration in cord plasma increases with increasing gestational age and is significantly higher than paired maternal values [11, 12]. With the abrupt termination of calcium transport across the placenta at delivery, plasma calcium falls, reaching a nadir at age 24–48 h [11]. Serum parathyroid hormone (PTH) increases postnatally in response to this fall in plasma calcium concentration. This increase in PTH mobilizes calcium from bone, and plasma calcium concentration rises and subsequently stabilizes even in the absence of exogenous calcium intake. Clinically significant hypocalcaemia occurs in asphyxiated newborns [12]. The etiology behind this is a sluggish response in PTH secretion to the postnatal fall in plasma calcium concentration.

When a neonate suffers asphyxia, series of clinical [13] and biochemical [14] alterations occur which can adversely affect the outcome [15]. While treating hyponatremic seizures, correction of the electrolyte disturbance is more effective than using anticonvulsants [16]. Hyperkalemia is associated with cardiac dysfunction and death. Hypocalcaemia is associated with jitteriness, cardiac dysfunction and seizure. Further the degree of electrolyte imbalance may vary according to the severity of birth asphyxia.

In a case control study by Basu P et al. among asphyxiated newborn, hyponatremia and hypocalcaemia developed early and simultaneously and the decrease in their serum levels was directly proportional to each other and to the degree of asphyxia among cases [17]. Similarly in a prospective study done by Shah G S et al. among asphyxiated neonates, hyponatremia and hypocalcaemia was noted respectively as 23.3 and 11.7% [18]. But in a case control study by Varma V et al. among asphyxiated newborns, mean values of electrolytes showed no significant difference among cases and controls as well as in HIE stages [19]. So there is limited literature regarding electrolyte disturbance in asphyxiated newborn especially the correlation with severity of asphyxia. So this study was started with the aim to study electrolyte (sodium, potassium, calcium) disturbances in asphyxiated newborns of different severity in the early neonatal period and to find out correlation of levels of sodium, potassium, calcium with severity of perinatal asphyxia.

## Methods

This prospective observational study was conducted in the Department of Paediatrics and Adolescent medicine, Bishweshwar Prasad koirala Institute of Health Sciences (BPKIHS), Dharan, Nepal during a study period of one year from June 2015 to May 2016 in asphyxiated new-borns born at this institute and a total of 88 cases were enrolled in the study.

### Aims

 ❖ To study electrolyte (sodium, potassium, calcium) disturbances in asphyxiated newborns of different severity in the early neonatal period.

❖ To find out correlation of levels of sodium, potassium, calcium with different severity of perinatal asphyxia.

#### Inclusion criteria

Term newborns born and admitted at BPKIHS & appropriate for gestational age (those babies falling between 10th to 90th percentile of weight for their gestational age i.e. weight between 2.5 to 4 kg) with Birth asphyxia as per WHO definition- “failure to initiate and sustain breathing at birth” and based on Apgar score as an Apgar score of < 7 at 5 min of life even after receiving resuscitation according to Neonatal Resuscitation Program (NRP) guidelines were included in the study.

#### Exclusion criteria

Preterm and IUGR (intrauterine growth retardation) babies, babies with gross congenital malformations, suspected metabolic diseases, cases receiving medications except vitamin K prior to collection of blood samples, those born to mothers with diabetes mellitus, mothers on antiepileptic, mothers with suspected or confirmed electrolyte abnormalities were excluded from the study.

Those born to mothers treated with diuretics, general anesthesia, phenobarbitone, pethidine, magnesium sulphate, antihypertensive and drugs likely to cause depression and electrolyte disturbance in newborn and parents not giving consent were also excluded from the study.

### Methodology

This was a hospital based prospective observational study. Consecutive sampling method was used for collection of sample. Apgar score at 1 and 5 min was taken after birth and cases were selected applying inclusion and exclusion criteria. Detailed antenatal, natal and postnatal history and clinical examination was done and findings were recorded on predesigned pro forma after informed assent obtained from parents. Relevant investigation as per protocol of BPKIHS were sent example- Complete Blood Count (CBC), Hematocrit, electrolytes (sodium, potassium, calcium), urea, creatinine, septic screen Total Leucocyte Count (TLC), absolute neutrophil count (ANC), band cell ratio, Micro Erythrocyte Sedimentation Rate (micro ESR), C-Reactive Protein (CRP) were sent from venous sampling within one hour of birth.

#### Electrolyte estimation

Serum electrolytes (sodium, potassium and calcium) was analyzed using ion selective electrode by automated machine.

#### HIE staging

Patients were classified according to Levene staging to grade the severity of HIE [20] (Table 1).

**Table 1:** A clinical grading system for hypoxic ischemic encephalopathy by LEVENE stage

Feature	Mild	Moderate	Severe
Consciousness	Irritable	Lethargy	Comatose
Tone	Hypotonia	Marked hypotonia	Severe hypotonia
Seizures	No	Yes	Prolonged
Sucking/Respiration	Poor suck	Unable to suck	Unable to sustain spontaneous respiration

No cases were treated with therapeutic hypothermia due to the unavailability of such facility at our institute and cases were managed according to the protocol of the institute. Normal level of serum sodium, potassium and ionized calcium was taken as 130-145 meq/l, 3.7–5.9 meq/l and 1–1.5 mmol/l respectively [21].

### Statistical analysis

Descriptive statistics was used for representation of data. The statistical data analysis was done using SPSS [Statistical Package for the Social Sciences (SPSS Inc.)] version 20.

Statistical comparison of mean values of different electrolytes with different severity of perinatal asphyxia was performed by ANOVA test for parametric data and significant data were further analyzed using post hoc test.

Bivariate analysis was done to determine the correlation between Apgar score at 5 min and serum electrolytes. Pearson test was used to calculate the correlation coefficient. Box plot was used to show median and quartiles of serum electrolytes with respect to Apgar score at 5 min.

## Results

Out of 88 enrolled cases 60 (68%) were male and 28 were (32%) female and mean weight being 2975.45 ± 349.53 g. (Tables 2 and 3).

**Table 2:** Electrolyte status according to different stages of HIE

Levene	No.	Hyponatremia	Hyperkalemia	Hypocalcaemia
Mild	25	0	1 (4%)	7 (28%)
Moderate	30	9 (30%)	16 (53.3%)	7 (23.3%)
Severe	33	27 (81.8%)	30 (90.9%)	15 (45.4%)

**Table 3:** Electrolyte characteristics

Electrolyte	Levene	No.	Mean	Std. deviation
Na	Mild	25	135.52	4.51
	Mod.	30	130.7	2.58
	Severe	33	127.15	2.26
	Total	88	130.73	4.60
K	Mild	25	4.96	0.73
	Mod.	30	5.93	0.55
	severe	33	6.78	0.87
	Total	88	5.98	1.03
Ca^++^	Mild	25	1.07	0.14
	Mod.	30	1.12	0.13
	severe	33	0.99	0.12
	Total	88	1.05	0.14

On comparing the means of sodium, potassium and ionized calcium between different stages of HIE using ANOVA, there was significant difference between them with *p*-value < 0.001 (Table 4).

**Table 4:** Post hoc test between two groups of HIE

Electrolyte	Levene	Levene	Mean Difference (A-B)	significance	95% confidence interval	
					Lower bound	Upper bound
Na	mild	Mod.	4.820	0.001	2.779	6.861
		severe	8.368	0.001	6.37	10.367
	Mod.	severe	3.548	0.001	1.647	5.45
K	mild	Mod.	−0.968	0.001	−1.445	−0.492
		severe	−1.816	0.001	−2.284	−1.35
	Mod.	severe	−0.848	0.001	−1.292	−0.404
Ca^++^	mild	Mod.	−0.048	0.382	−0.134	0.038
		severe	0.080	0.066	−0.004	0.165
	Mod.	severe	0.128	0.001	0.048	0.209

Bivariate analysis was used to find the correlation between Apgar score and serum electrolyte level and Pearson test was used to find correlation coefficient. Box plot was used to show median and quartiles of serum electrolyte with respect to Apgar at 5 min.

**Fig. 1:**
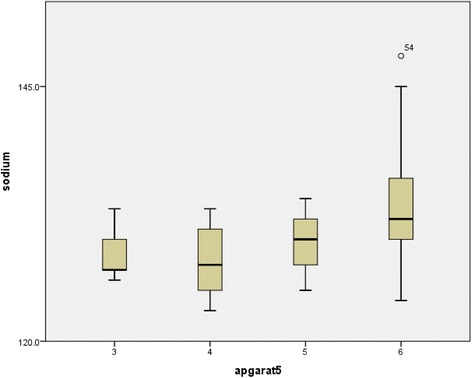
Box plot showing median and quartiles of serum sodium at 5 min Apgar

Significant correlation was present between serum sodium and Apgar score at 5 min with *p*-value < 0.001 i.e. as Apgar score at 5 min increased serum sodium also increased with Pearson correlation co-efficient of 0.448 (Fig. 1).

**Fig. 2:**
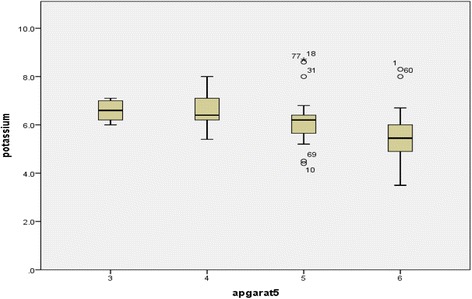
Box plot showing median and quartiles of serum potassium at 5 min Apgar

There was significant negative correlation between serum potassium and Apgar at 5 min with p-value < 0.001 i.e. as Apgar score at 5 min increased serum potassium decreased with Pearson correlation coefficient of − 0.422 (Fig. 2).

**Fig. 3:**
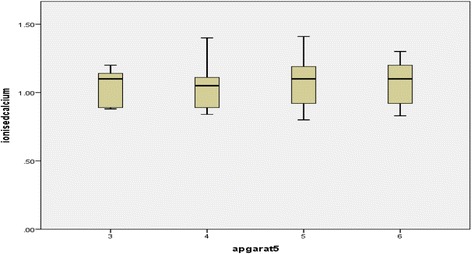
Box plot showing median and quartiles of serum ionized calcium at 5 min Apgar

No significant correlation was present between Apgar at 5 min and serum ionized calcium level with p-value< 0.077 i.e. change in the value of Apgar score at 5 min had no relation with change in serum ionized calcium level and Pearson correlation coefficient of 0.479 (Fig. 3).

## Discussion

The overall mean of sodium, potassium and ionized calcium was 130.73 ± 4.60 meq/l, 5.98 ± 1.03 meq/l and ionized calcium 1.05 ± 0.14 mmol/l respectively. In a case control study done by Basu P et al., the mean value of sodium, potassium and calcium was 122.1 ± 6.0 meq/l, 5.05 ± 0.63 meq/l and calcium 6.85 ± 0.95 respectively [17]. Similarly in another case control study done by Varma V et al., the means of sodium and potassium among cases were 136.5 ± 8.7 meq/l and 3.78 ± 0.36 meq/l respectively [19]. The difference between the results were probably because of difference in the timing of collection of samples, as we collected blood sample as early as possible no later than one hour of life, so chance of correction of electrolyte by body’s internal milieu was less.

Basu et al. [17] found increased severity of hyponatremia, hyperkalemia and hypocalcaemia with increased severity of birth asphyxia. The pattern of hyponatremia and hyperkalemia was similar to our study. Similarly in case control study by Jajoo et al. [22], Rai [23] et al. and Schedewie et al. [24], showed that asphyxiated newborns had lower serum calcium level compared to their controls.

The treatment of hyponatremia in such condition is by fluid restriction rather increasing sodium load for reasons mentioned in background section. So fluid should be restricted in cases of birth asphyxia till normalization of serum sodium with close monitoring of weight and serum sodium. Serum potassium and Electrocardiography (ECG) monitoring should be done to avoid the deadly complications of hyperkalemia. Apart from other treatment measures, correction of acidosis and use of potassium free fluid are the most useful measures to correct hyperkalemia. Our study however did not find significant hypocalcaemia with increasing severity of HIE but there was hypocalcaemia associated with birth asphyxia, so regular supplementation and monitoring of serum calcium should be done.

Our study had various limitations:

1. Our classification of HIE was according to Levene stage which is simple but it doesn’t take various parameters into consideration like EEG.
2. Intravenous fluid and oxytocin used in pregnant women might affect the electrolyte status in mother and hence in newborn was not taken into consideration.
3. Blood pH was not taken into consideration which can affect serum potassium level.

## Conclusion

Hyponatremia, hyperkalemia and hypocalcaemia occur in neonates with birth asphyxia which may cause increased morbidity and mortality. More severe hyponatremia should be suspected if there is severe birth asphyxia and vice versa. Hence its level should be more regularly monitored to prevent the problems associated with it. Severe hyperkalemia is associated with severe birth asphyxia and vice versa; so regular potassium monitoring and ECG monitoring is required to detect cardiac changes associated with it so that prompt management can be instituted.

## Abbreviations

AAP: American Academy of Pediatrics
ADH: Anti-diuretic Hormone
ANC: Absolute Neutrophil Count
ANOVA: Analysis of Variance
ARF: Acute Renal Failure
Ca^++^: Ionized calcium
CBC: Complete Blood Count
CRP: C-Reactive Protein
CT: Collecting Tubules
ECG: Electrocardiogram
ECHO: Echocardiogram
ESR: Erythrocyte Sedimentation Rate
HIE: Hypoxic Ischemic Encephalopathy
ICU: Intensive Care Unit
IEC: Institutional Ethical Committee
IUGR: Intrauterine Growth Retardation
K: Potassium
Na: Sodium
NRP: Neonatal Resuscitation Program
PIH: Pregnancy Induced Hypertension
POG: Period of Gestation
PTH: Parathyroid Hormone
SIADH: Syndrome of Inappropriate Antidiuretic Hormone Secretion
SPSS: Statistical Package for Social Sciences
TLC: Total Leucocyte Count
WBC: White Blood Count
WHO: World Health Organization
